# Assessing the effects of interventions for *Aedes aegypti* control: systematic review and meta-analysis of cluster randomised controlled trials

**DOI:** 10.1186/s12889-017-4290-z

**Published:** 2017-05-30

**Authors:** Víctor Alvarado-Castro, Sergio Paredes-Solís, Elizabeth Nava-Aguilera, Arcadio Morales-Pérez, Lidia Alarcón-Morales, Norma Alejandra Balderas-Vargas, Neil Andersson

**Affiliations:** 1 Centro de Investigación de Enfermedades Tropicales (CIET), Universidad Autónoma de Guerrero, Acapulco, Guerrero Mexico; 2 Unidad Académica de Matemáticas, Universidad Autónoma de Guerrero, Chilpancingo, Guerrero Mexico; 3 Department of Family Medicine, McGill University, Montreal, Canada

**Keywords:** Dengue, chemical control, biological control, community mobilisation, meta-analysis

## Abstract

**Background:**

The *Aedes aegypti* mosquito is the vector for dengue fever, yellow fever, chikungunya, and zika viruses. Inadequate vector control has contributed to persistence and increase of these diseases. This review assesses the evidence of effectiveness of different control measures in reducing *Aedes aegypti* proliferation, using standard entomological indices.

**Methods:**

A systematic search of Medline, Ovid, BVS, LILACS, ARTEMISA, IMBIOMED and MEDIGRAPHIC databases identified cluster randomised controlled trials (CRCTs) of interventions to control *Aedes aegypti* published between January 2003 and October 2016. Eligible studies were CRCTs of chemical or biological control measures, or community mobilization, with entomological indices as an endpoint. A meta-analysis of eligible studies, using a random effects model, assessed the impact on household index (HI), container index (CI), and Breteau index (BI).

**Results:**

From 848 papers identified by the search, eighteen met the inclusion criteria: eight for chemical control, one for biological control and nine for community mobilisation. Seven of the nine CRCTs of community mobilisation reported significantly lower entomological indices in intervention than control clusters; findings from the eight CRCTs of chemical control were more mixed. The CRCT of biological control reported a significant impact on the pupae per person index only. Ten papers provided enough detail for meta-analysis. Community mobilisation (four studies) was consistently effective, with an overall intervention effectiveness estimate of −0.10 (95%CI -0.20 – 0.00) for HI, −0.03 (95%CI -0.05 – -0.01) for CI, and −0.13 (95%CI -0.22 – -0.05) for BI. The single CRCT of biological control had effectiveness of −0.02 (95%CI -0.07– 0.03) for HI, −0.02 (95%CI -0.04– -0.01) for CI and −0.08 (95%CI -0.15– -0.01) for BI. The five studies of chemical control did not show a significant impact on indices: the overall effectiveness was −0.01 (95%CI -0.05– 0.03) for HI, 0.01 (95% CI -0.01– 0.02) for CI, and 0.01 (95%CI -0.03 – 0.05) for BI.

**Conclusion:**

Governments that rely on chemical control of *Aedes aegypti* should consider adding community mobilization to their prevention efforts. More well-conducted CRCTs of complex interventions, including those with biological control, are needed to provide evidence of real life impact. Trials of all interventions should measure impact on dengue risk.

## Background

In 2013, Bhatt and colleagues estimated 390 million dengue infections worldwide each year, with 96 million of these producing some clinical manifestation [1]. They estimated that Asia accounts for 70% of these infections, India alone accounting for 34%; 14% occur in the Americas, more than half of which occur in Brazil and Mexico; 16% occur in Africa, and only 0.2% in Oceania [1]. Since publication of the articles in this review, a new dengue vaccine has been approved for use in Mexico [2], the Philippines [3] and Brazil [4]. Notwithstanding the new vaccine, vector control probably will remain an important element of dengue prevention and dengue prevention research [5, 6]. A World Health Organisation (WHO) meeting of experts in March 2016 noted, however, that there was no evidence that recent vector-control efforts such as massive use of insecticides have a significant effect on dengue transmission [7].

*Aedes aegypti* is an important vector for dengue virus infection. Apart from dengue virus, *Aedes aegypti* is also the vector for transmission of other viruses presenting serious public health threats: chikungunya [8, 9], zika [10] and yellow fever [11]. There is currently no vaccine available for chikungunya or zika. Following a big outbreak of zika in Brazil, including cases of microcephaly among babies born to infected mothers, WHO declared zika a public health emergency of international concern and issued a response framework and operations plan for tackling zika worldwide [12]. There is a huge shortfall in funding for the WHO response programme [13]; with limited funding there is an urgent need to identify the most effective interventions for *Aedes aegypti* vector control.

Summarised in Table 1, 12 systematic reviews synthesized evidence of the effectiveness of chemical, biological and community participation interventions for control of the *Aedes aegypti* vector and dengue infection [14–25]. These covered 278 studies with considerable overlap, including 26 cluster randomised controlled trials (CRCTs). The most common study design was a non-randomised controlled trial (110 studies), and before-after analysis (88 studies). Some reviews had a broad focus, covering multiple interventions [15, 17, 24, 25], others covered more specific community-based interventions [14, 19] or outbreak control [16]. Some were limited to single specific interventions, such as peridomestic spraying of insecticide [18], use of *Bacillus thuringiensis israelensis* [20], temephos [21], larvivorous fish [22] or copepods [23].

**Table 1:** Summary of systematic reviews on dengue vector control from 2007 to 2016

Author and year	Focus of the review	Number of studies	Epidemiological design of the included studies	Main conclusions
Heintze (2007) [14]	Community-based dengue control interventions	11	2 Randomized controlled trials 3 Interrupted time series 6 Before-after analysed trials	Interventions and outcomes varied. Six studies combined community participation programmes with dengue control tools. Only 2 papers reported confidence intervals; 5 reported *p*-values; none were cluster randomized. Weak evidence that community-based programmes alone or in combination can enhance dengue control.
Erlanger (2008) [15]	Effect of different dengue control methods on entomological indices in developing countries. (With meta-analysis)	56	2 Cluster randomized control trials 2 Randomized controlled trials 23 Non-randomized controlled trials 2 Interrupted time series 24 Before-after analysed trials 3 Observational studies	Integrated vector management most effective method to reduce CI, HI and BI. Environmental management alone relatively low effectiveness. Biological control targeted small numbers; IVM targeted larger populations. Most effective is a community-based, integrated approach, tailored and combined with educational programmes.
Pilger (2008) [16]	Response to dengue outbreaks	24	4 Non-randomized controlled trials 2 Interrupted time series 4 Before-after analysed trials 14 Observational studies	Combined interventions of vector control (community involvement & use of insecticides), training of medical personnel, plus laboratory support, helped control outbreaks. Spatial spraying of insecticides alone ineffective and its usefulness with other interventions is doubtful.
Ballenger-Browning (2009) [17]	Impact of biological, chemical and educational interventions on entomological indices	21	2 Cluster randomized control trials 3 Randomized controlled trials 3 Interrupted time series, 13 Non-randomized controlled trials	Evidence of efficacy lacking: poor study designs and lack of congruent entomologic indices. Need more cluster randomized controlled trials.
Esu (2010) [18]	Effect of peridomestic insecticide spraying on dengue transmission	15	1 Cluster randomized control trial 14 Before-after analysed trials	Few studies of effectiveness of peri-domestic space spraying. Best applied as part of IVM. Need to measure impact of spraying on adult and immature mosquitoes and disease transmission.
Al-Muhandis (2011) [19]	Impact of educational messages and community based approach (With meta-analysis)	21	3 Cluster randomized control trials 4 Non-randomized controlled trials 14 Before-after analysed trials	Important impact of educational messages in a community-based approach on larval indices. Very heterogeneous effect size with different study designs; interpretation of pooled results difficult.
Boyce (2013) [20]	*Bacillus thuringiensis israelensis* (Bti) for the control of dengue vectors	14	2 Cluster randomized control trials 1 Randomized controlled trial 11 Non-randomized controlled trials	Bti can reduce the number of immature *Aedes* in the short term, but very limited evidence that Bti alone can reduce dengue morbidity. Need to measure impact of Bti in combination with other strategies to control dengue vectors.
George (2015) [21]	Community effectiveness of temephos for dengue control	27	3 Cluster randomized control trials 11 Non-randomized controlled trials 13 Before-after analysed trials	Temephos alone suppressed entomological indices; did not do so when combined with other interventions. No evidence that temephos use is associated with reduced dengue transmission.
Han (2015) [22]	Efficacy and community effectiveness of larvivorous fish for dengue vector control	13	9 Non-randomized controlled trials 4 Before-after analysed trials	Larvivorous fish alone or combined with other control measures may reduce immature vector stages. Study limitations preclude conclusions about community effectiveness. Need cluster randomised controlled trials with measurement of impact on dengue transmission
Lazaro (2015) [23]	Community effectiveness of copepods for dengue vector control	11	11 Non-randomized controlled trials	Limited evidence of impact of cyclopoid copepods as a single intervention. Very few studies; more needed in other communities and environments.
Lima (2015) [24]	Impact of chemical, physical and biological control (With meta analysis)	26	6 Cluster randomized control trials 16 Non-randomized controlled trials, 4 Before-after analysed trials	The most effective control method was IVM, starting with community empowerment as active agents of vector control.
Bowman (2016) [25]	Effectiveness of different control methods, alone and in combination, on vector indices and dengue transmission (With meta analysis)	39	7 Cluster randomized control trials 2 Randomized controlled trials 8 Non-randomized controlled trials 11 Interrupted time series 5 Before-after analysed trials 6 Observational studies	Lack of reliable evidence on the effectiveness of any dengue vector control method. High quality studies (such as CRCTs) are needed, with measurement of effect on dengue transmission as well as vector indices.

Total of 278 studies reviewed (with considerable overlap): 26 CRCTs; 10 RCTs; 110 non-randomised controlled trials; 21 interrupted time series; 88 before-after analyses; 23 observational studies

Several reviews concluded that some form of integrated vector management (IVM), including chemical control, community involvement, and co-operation between services was the best approach to reduce entomological indices of *Aedes aegypti* infestation or control outbreaks of dengue [15, 16, 24]. WHO recommends IVM for control of vector borne diseases, including dengue [26, 27].

The authors of many of the previous reviews noted that their conclusions were limited by the poor quality of the available evidence. Existing evidence studied impact mostly on vector indices rather than on dengue infection or disease incidence. While reviews suggested effectiveness of community involvement and mobilisation, the weak study designs and poor quality of reporting made interpretation difficult [14, 19]. Reviews focusing on specific biological control methods were largely unable to conclude about effectiveness because the relatively few published studies generally had weak designs [20, 22, 23]. Reviews of specific chemical interventions were also limited in their conclusions. A review of 15 studies of peridomestic insecticide spraying included only one CRCT, the remainder using before-after analyses [18]. A review of 27 studies of the effectiveness of temephos for dengue control included only three CRCTs; the authors concluded there was evidence that temephos alone, although not in combination, suppressed entomological indices, but noted there was no evidence that temephos use was associated with decreased dengue transmission [21]. Authors of a 2009 review including multiple approaches for dengue control complained of the problems of poor study design and non-comparable entomological endpoints [17], and a recent review of the effects of multiple dengue prevention approaches noted a lack of reliable evidence of effectiveness, particularly on the endpoint of dengue incidence [25].

Review authors have repeatedly called for more cluster randomised controlled trials of single and combined interventions for dengue prevention, with measurement of their impact on dengue transmission as well as on vector indices [17, 22, 25]. The aim of the present study is to review the effectiveness of interventions for dengue vector control, specifically as measured in CRCTs. This limits the number of eligible studies, but means that the findings of those that are included are likely to be more reliable.

## Methods

### Search strategy

In 2013 we carried out a systematic search for articles published between January 2003 and June 2013 assessing the impact of chemical control, biological control and/or community mobilization as strategies for *Aedes aegypti* vector control. We searched the Medline, Ovid, BVS, LILACS, ARTEMISA, IMBIOMED and MEDIGRAPHIC databases. The search terms we used were “dengue”, “*Aedes aegypti*”, “chemical control”, “biological control”, “community-based”, “community mobilisation”, “social mobilisation”, “community empowerment”, “effectiveness” and “vector control”, and their Spanish and Portuguese equivalents. We updated the search in November 2016 to cover articles published up to the end of October 2016. We also reviewed the references listed in identified publications and included additional studies found in these lists, limiting our search to publications in English, Spanish or Portuguese.

**Fig. 1:**
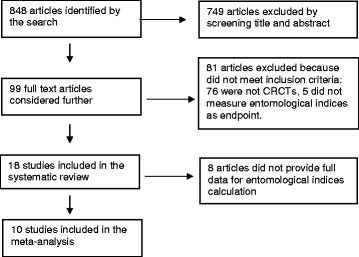
Flow chart of studies included in the meta-analysis

Figure 1 is a flow chart of the studies identified and finally included in the systematic review and meta analysis. The first search in 2013 produced a list of 588 articles. In 2015, we added a further 27 studies and in 2016 we added a further 233 studies identified by a new electronic search and a manual search (total 848 articles). Two reviewers (VA and LA), working independently, reviewed the title and abstract of these articles. They excluded 749 articles: 590 because they clearly did not meet the inclusion criteria, and 159 because they were further publications of the same studies.

The pre-established inclusion criteria were:

- studies concerned directly with the impact of chemical control, biological control or community mobilisation, alone or in combination, on dengue vector parameters;
- studies that were cluster randomized controlled trials; and
- studies that provided information about at least one of the three standard *Aedes aegypti* indices: house index (HI) -- households with larvae or pupae as a proportion of households examined; container index (CI) -- containers with larvae or pupae as a proportion of containers examined; and Breteau index (BI) -- containers with larvae or pupae as a proportion of households examined.

The reviewers read the full text of the remaining 99 candidate articles and excluded 81 of them as not meeting the inclusion criteria. Five of the excluded studies did not measure impact on entomological indices, and 76 were not CRCTs. This systematic review includes all 18 remaining articles; 10 of these had the necessary information for calculation of the entomological indices to allow us to include them in the meta-analysis.

### Data extraction and quality assessment

We extracted data from the 18 articles using a format developed by consensus among study team members. Two reviewers extracted the data independently and then resolved discrepancies by consensus. We assessed methodological validity of the studies using the Cochrane approach for assessing the risk of bias [28]. This includes an assessment of how the studies handled and reported: random sequence generation, blinding of participants and personnel, blinding of outcome assessment, handling of incomplete data, and selectiveness of reporting. We graded each paper for each domain as having low, unclear or high risk of bias, and then calculated an overall risk of bias.

### Meta-analysis

We defined intervention effectiveness for each of the entomological indices (HI, CI and BI) as the difference between the intervention group and the control group at the last point of measurement. For each type of intervention (chemical control, biological control, community mobilisation) we performed a meta-analysis using a random effects model to estimate global intervention effectiveness for each entomological index (HI, CI, BI), estimating the combined overall Risk Difference (RD) and its 95% CI. The model took into account inter- and intra-study variability by weighting [29]. We carried out the analysis using the open-source software CIETmap [30] and the “*meta*” package of the statistical language R [31].

We performed the DerSimonian and Laird Q test [32] to assess the level of heterogeneity, with the null hypothesis of non-heterogeneity. We derived *p*-values for this test by comparing the Q statistic with the α-percentile of a χ^2^ distribution with k-1 degrees of freedom (where *k* is the number of studies).

For each type of intervention, we measured each study’s influence on the overall estimated intervention effectiveness by replicating the meta-analysis for each of the three entomological indices, eliminating one of the included studies from the analysis at each step. We then quantified the differences in the overall results [29, 33].

We assessed publication bias using a funnel plot, which shows the sample size of each study next to the detected effect size. We used the Begg and Egger statistical test [34, 35] and considered *p* < 0.10 to be a statistically significant indicator of publication bias.

## Results

Table 2 shows details of the 18 CRCTs that met our inclusion criteria for the review [36–53]. Published between January 2003 and December 2015, these studies all implemented interventions and measured impact at the cluster level. The 18 studies covered 246 intervention clusters (48,131 intervention households) and 288 control clusters (69,430 control households) in 13 countries: India, Thailand, Sri Lanka, Cuba, Haiti, Mexico, Guatemala, Nicaragua, Venezuela, Brazil, Uruguay, Ecuador and Colombia. Of the 18 CRCTs, we categorised eight as trials of chemical control interventions, one as a trial of a biological control method, and nine as trials of community mobilisation for dengue prevention.

**Table 2:** Interventions and main findings of the 18 cluster randomised controlled included in the systematic review

Author, year, country	Period	Intervention			Control			Indices & measurement	Main results	Conclusions of authors
		Actions	Clust	HH	Actions	Clust	HH			
Chemical control interventions										
Camargo (2002) Brazil [36]	Sep 2000 – Jun 2001	1% temephos applied to HH water containers 3 monthly. Community removal of “removable” water containers.	1	17,994	Community removal of “removable” water containers.	1	37,955	BI, CI measured monthly for 10 m in 300 HH randomly selected in both intervention and control clusters	BI and CI slightly lower in control cluster than intervention cluster at most time points. Both BI and CI were related to rainfall.	Not clear why temephos was not effective.
Kroeger (2006) Mexico and Venezuela [37] MA	Mexico Oct 2002 - Nov 2003 Venezuela Jan –Nov 2003	*Mexico* Window curtains with lambdacyhalothrin and pyriproxyfen chips in water. (HH took out chips; not considered further.) *Venezuela* Window curtains and water jar covers treated with longlasting deltamethrin	18	1116	Control clusters received no interventions	18	1108	HI, CI, BI, PPI measured at baseline, 4w, 4 m & 12 m (Mexico) 9 m (Venezuela). Adult dengue IgM serology at baseline & 8 m in Venezuela in approx 650 HH	At last measurement in Mexico and Venezuela, no significant difference in entomological indices between intervention and control clusters; but indices in all clusters significantly lower than at baseline. No such fall in nearby “external control” sites. Levels of dengue IgM lower in intervention clusters than control clusters (*p* = 0.06)	The fall in control sites was due to spill-over effect. Insecticide treated curtains can reduce dengue vector levels and potentially dengue transmission
Lenhart (2008) Haiti [38] MA	Jul 2003 – Jan 2004	Insecticide (permethrin) treated bednets (ITNs) supplied to households	9	495	No treatment for 5 months; received ITNs after 6 months	9	522	BI, HI, CI, PPI measured at baseline, 1 m, 5 m and 12 m. IgM dengue serology measured at 12 m	At 1 m, all indices fell in the ITN sites. By 5 m, indices had also fallen in control sites and were now lower than in ITN sites. Control HH near to ITN sites had lower indices. At 12 m all sites had significantly lower indices and fewer IgM positives than at baseline.	Lack of difference between ITN and control sites due to spill-over. ITNs can reduce vector indices and potentially dengue transmission.
Ocampo (2009) Colombia [39] MA	Apr 2004 – Jul 2005	In 3 intervention clusters used a. Lethal Ovitraps with deltamethrin (LO), b. *Bacillus thuringiensis israelensis* briquettes (Bti) and c. LO + Bti Initial education of HH about dengue and vector breeding and bi-weekly visits of research team	3	240	Initial education of HH about dengue and vector breeding and bi-weekly visits of research team	1	80	HI, PHI, adult index measured at baseline and twice monthly for 4 m in 10 HH for each intervention cluster (total 30 HH) and 10 control HH	No difference between intervention clusters and the control cluster in any indices. Control cluster indices not different from indices from all clusters measured in previous year, but those in all three intervention clusters combined were lower than those in the previous year.	Lack of difference between intervention and control clusters suggests initial education and repeated visits were as effective as the interventions. Small sample size was an issue.
Rizzo (2012) Guatemala [40]	Mar 2009 -Oct 2010	*Intervention 1* Window & door nets treated with deltamethrin and water container covers treated with deltamethrin (wrong size so not used). Govt programme treated water with 1% temephos in 3 intervention and 3 control clusters. *Intervention 2* After 17 months, nets replaced as needed and “productive” containers treated with temephos or discarded. Govt programme continued as above	10	970	Govt programme treated water with 1% temephos in 3 intervention and 3 control clusters	10	865	At baseline, 6w after first intervention and 6w after second intervention measured total pupae, PPI, HI, BI and CI.	6w after first intervention, indices higher in all clusters than baseline. Total pupae and PPI increased more in control clusters but difference not significant. 6w after second intervention, indices were lower overall. Total pupae reduced significantly more in intervention clusters, borderline difference for PPI. HI reduced significantly more in intervention clusters, borderline difference for BI and no significant difference for CI.	The combination of insecticide treated curtains and targeting productive container types (with temephos and discarding containers) can reduce the dengue vector population.
Vanlerberghe (2013) Thailand [41] MA	Oct 2007 -Sep 2008	Window and door nets treated with long-lasting deltamethrin formulation. Max 5 nets/HH. Insecticide supposed to last two years. Routine government vector control.	22	2032 (80–110 hh/cluster)	Routine government vector control including temephos available to HH and deltamethrin spraying if case of dengue detected.	66	660 (10 hh/cluster)	BI, HI, CI and PPI measured at baseline, 6 m & 18 m. All HH in control clusters; random half of HH in intervention clusters	At 6 m, BI was significantly lower in intervention clusters. HI, CI and PPI were also lower in intervention clusters. At 18 m, BI was no longer lower in intervention clusters, and nor were HI, CI and PPI. At 6 m, 71% of HH used the nets, but only 33% used them at 18 m.	Insecticide treated window and door nets can reduce vector breeding. The effect is coverage dependent.
Quintero (2015) Colombia [42] MA	Jul 2013 – Mar 2014	*Immediate:* Window and door nets treated with deltamethrin in all 10 clusters. *After 8 m in 4 clusters*: Water container covers treated with deltamethrin. Routine government vector control activities continued.	10	922	Routine government vector control activities: temephos in water containers, health education, occasional malathion space spraying.	10	891	Measured at baseline, 9w after first intervention, and 4-6w after second intervention: CI, HI, BI, PPI	At first follow up indices fell more (cf baseline) in intervention clusters than control clusters; I-C difference significant for BI only. PPI *increased* in intervention clusters. At second follow up all indices including PPI decreased more in intervention clusters; significant by t-test but not by non-parametric test.	The intervention package can reduce dengue vector density. Needs behaviour change for sustained effect.
Che-Mendoza (2015) Mexico [43]	Mar 2011 –Oct 2013	Door and window screens treated with alpha-cypermethrin. After 14 m, productive containers also treated with spinosad every 2 m. Routine government vector control continued.	10	1000	Routine government vector control: temephos in water containers, space spraying with chloropyrifos and propoxur.	10	1000	Measured BI, CI, HI & PPI at baseline and at 5, 12, 18 and 24 m Also measured adult mosquitoes.	Only adult mosquitoes less in intervention HH after the treated screens. At 18 m (after treatment of productive containers), BI, CI, HI and PPI significantly lower in the intervention clusters. At 24 m, only PPI significantly lower.	Insecticide treated screens & treatment of productive containers with spinosad can reduce vector breeding for up to 24 m
Biological control interventions										
Kittayapong (2012) Thailand [44] MA	May –Nov 2010	Community mobilisation meetings and recruitment of ecohealth volunteers. Either copepods or *Bacillus thuringiensis israelensis* toxin (Bti) to HH water containers, plus screen net covers for containers. Education about dengue vector by ecohealth volunteers	10	441	No intervention	10	448	HI, CI, BI & PPI measured at baseline, 2 m, 4 m and 6 m.	Vector indices lower in all clusters than at baseline. No significant difference between intervention and control clusters in HI, CI, BI. PPI was significantly lower in intervention than control clusters at 2 m, 4 m & 6 m	It was feasible to implement the intervention in urban and peri-urban settings. Reduced the vector density (as judged by PPI)
Community participation and community mobilisation interventions										
Espinoza-Gomez (2002) Mexico [45]	Sep 1998 –Apr 1999	a. *Education*. House visits by university students, educational materials (eg calendars), group meetings with video + sociodrama (47 HH) b. *Chemical*. ULV spraying malathion & temephos to water containers (46 HH) c. *Education & chemical* (49 HH)	3	142	d. *Control.* No intervention (45 HH)	1	45	Baseline and 6 m. Measured BI, CI, HI and positive containers/HH (C+/H).	Reported on C+/H only. Reduced from baseline to 6 m in Education only cluster, but not in Chemical or Control clusters. In the Education & chemical cluster, reduction from baseline was less marked.	Education intervention was effective but Chemical intervention was not. The Chemical intervention reduced the effect of the Education intervention, perhaps by false sense of security.
Vanlerberghe (2009) Cuba [46] MA	Jan 2005 –Feb 2006	>Stakeholder discussions, steering committee >Community working groups, action plans >Coordination between community and services >Harmonisation with local vector control plan. Government routine vector control programme continued.	16	8422	Government routine vector control programme: House inspections, temephos to water containers, space spraying with cypermethrin or cloripyriphos, health education, fines for law infringements	16	10,748	HI, BI and PPI measured at several points between baseline and end at 15 m	The HI, BI and PPI were not different between intervention and control clusters at baseline. At 15 m, HI, BI and PPI were all significantly lower in intervention clusters compared with control clusters.	A community based environmental management strategy on top of routine programme reduced dengue vector indices.
Arunachalam (2012) India [47] MA	Jun 2009 – Dec 2010	>Stakeholder consultation meetings >Involvement of women self-help groups >Mobilisation of schools, teachers & schoolchildren >Communities distributed locally-made container covers and educational materials. Routine government control services.	10	1000	Routine government control services only. Some of the trial educational materials	10	1000	CI, BI, HI and PPI measured at baseline, 5 m and 10 m	At 10 m there were significant reductions in the HI, BI, CI and PPI in the intervention vs control clusters.	A community-based approach involving multiple stakeholders to implement control actions reduced dengue vector indices.
Abeyewickreme (2012), Sri Lanka [48]	Feb 2009 – Feb 2010	>Building partnerships of local stakeholders >Household solid waste management promoted by HH volunteers >Promoting composting of biodegradable waste >Improvement of local gov rubbish collection	4	803	Local government services	4	790	Measured PPI, HI, CI and BI at baseline, 3 m, 9 m and 15 m	No significant differences between intervention and control clusters for HI, CI. BI significantly lower at 15 m. PPI significantly reduced in intervention clusters.	Household and community involvement helped reduce solid waste containers which are major site of dengue breeding.
Castro (2012) Cuba [49]	Oct 2004 –Dec 2007	Participatory strategy: > Organisation and management structures > entomological risk surveillance >capacity building at local & intermediate level >community work in vector control, led by community working groups (CWGs) who visited HH, planned actions Government routine vector control programme continued	16	389	Government routine vector control programme: House inspections, temephos to water containers, space spraying with cypermethrin or cloripyriphos, health education, fines for law infringements	16	390	BI measured monthly from government surveillance figures before and during intervention from mid 2005 to Dec 2007.	Over the intervention period, the BI remained significantly lower in the intervention clusters than in the control clusters; the difference was bigger after the CWGs began their activities.	The empowerment strategy increased community involvement and added effectiveness to routine vector control.
Caprara (2015) Brazil [50]	Jun 2012 – May 2013	>Community workshops >Mobilising elders and schoolchildren for solid waste management >Government workers encouraged covering water containers >Educational materials	10	1689	Routine government vector control programme.	10	1580	HI, CI, BI, PPI measured at baseline and 6 m	All indices significantly lower in the intervention clusters at 6 m.	Social participation and environmental management is feasible and significantly reduced vector indices.
Mitchell-Foster (2015) Ecuador [51]	Nov 2012 – Nov 2013	An integrated intervention strategy (IIS) >Elementary school education programme >Clean Patio Safe Container programme with community volunteer activators	10	993	Government control programme: >Initially temephos and space spraying with insecticide >Midway, changed to biolarvicide (Bti) and HH education for source reduction	10	993	HI, BI and PPI measured at baseline and 12 m	PPI was significantly reduced in intervention clusters vs the control clusters (now with Bti) but only when clusters without full implementation were excluded.	Complicated by change in government programme midway through trial period. Need to explore integration of biolarvicide with the IIS approach.
Basso (2015) Uruguay [52] MA	Nov 2012 – Apr 2013	Campaign with community members & health institutions for removal of water containers around households (bags with containers collected). Engagement of community opinion makers, leaflets, & press conference.	10	1000	Routine removal of the containers by services	10	1000	BI, CI, HI, PPI & PHI measured at baseline and 5 m (1 m after intervention)	The increase in indices from dry to wet season was less in the intervention communities but the difference was not statistically significant.	Low vector densities meant sample size did not have sufficient power to detect differences as significant.
Andersson (2015) Nicaragua and Mexico [53] MA	Jul 2010 - Feb 2013	Community discussions of baseline evidence on vector breeding sites & infection in children. Community groups planned actions: HH visits by community brigades, school activities, & community clean-up activities and events. Government control programme continued.	75	9529	Government dengue control programme: temephos in HH water containers & peridomestic space spraying.	75	9309	HI, CI, BI, PPI & IgM dengue saliva serology measured at baseline, 12 m, and 15 m (Mexico) 17 m (Nicaragua)	All vector indices significantly lower in intervention than control clusters in follow up survey. Dengue infection rates in children aged 3–9 years (paired saliva samples) and self-reported dengue cases significantly lower in intervention than control sites.	Evidence based community mobilization effective for dengue vector control. Tailored implementation for individual sites gives local customization & strong community engagement.

*HH* = households, *HI* = household index; *CI* = container index; *BI* = Breteau index; *PPI* = pupae per person index; *PHI* = pupae per house index;

MA = study included in the meta-analysis

Table 3 shows the risk of bias assessments for the 18 studies. We assessed eight studies as having a low risk of bias overall, the remaining 10 having an unclear risk of bias mainly because they did not provide enough information to assess some elements of the risk of bias.

**Table 3:** Risk of bias assessment for the 18 studies, using Cochrane method

First author & year	Intervention	Blinding of participants & personnel	Blinding of outcome assessment	Incomplete outcome data	Selective reporting	Other sources of bias	Summary of risk of bias assessment^a^
Camargo (2002) [36]	Chemical control	1	2	2	2	2	2
Kroeger (2006) *MA* [37]	Chemical control	1	2	1	1	2	1
Lenhart (2008) *MA* [38]	Chemical control	1	2	2	2	2	2
Ocampo (2009) *MA* [39]	Chemical control	1	2	2	1	2	2
Rizzo (2012) [40]	Chemical control	1	2	2	1	2	2
Vanlerberghe (2013) *MA* [41]	Chemical control	1	2	1	1	2	1
Quintero (2015) *MA* [42]	Chemical control	1	2	1	1	2	1
Che-Mendoza (2015) [43]	Chemical control	1	2	2	1	2	2
Kittayapong (2012) *MA* [44]	Biological control	1	2	2	1	2	2
Espinoza-Gomez (2002) [45]	Community participation	1	2	2	1	2	2
Vanlerberghe (2009) *MA* [46]	Community participation	1	2	1	1	2	1
Arunachalam (2012) *MA* [47]	Community participation	1	2	1	1	2	1
Abeyewickreme (2012) [48]	Community participation	1	2	2	2	2	2
Castro (2012) [49]	Community participation	1	2	1	1	2	1
Caprara (2015) [50]	Community participation	1	2	2	1	2	2
Mitchell-Foster (2015) [51]	Community participation	1	2	1	1	2	1
Basso (2015) *MA* [52]	Community participation	1	2	2	1	2	2
Andersson (2015) *MA* [53]	Community participation	1	1	1	1	2	1

1 = Low risk of bias; 2 = Unclear risk of bias; 3 = High risk of bias.

^a^The summary figure is the median of the five individual elements

*MA* = Included in the meta-analysis

### Chemical control interventions

Among the eight CRCTs categorised as chemical control interventions, five tested the effect of insecticide-treated window and door screens or curtains: one as a single intervention [41], two combined with insecticide-treated water container covers [37, 42], and two combined with temephos or spinosad treatment of productive water containers [40, 43]. One trial tested the impact of insecticide-treated bed nets as a single intervention [38] and one tested the impact of temephos applied to water containers as a single intervention [36]. Ocampo et al. reported on a trial of lethal ovitraps and *Bacillus thuringiensis israelensis* (Bti) briquettes, alone or in combination, together with an initial education and clean-up campaign and regular household visits. Since education/clean-up and visits alone was also the ‘control’ condition, we categorised this as a chemical intervention of the deltamethrin lethal ovitraps [39]. Three trials had a staged intervention: in Guatemala deltamethrin-treated window and door nets were replenished and supplemented with temephos treatment of productive containers after 17 months [40]; in Colombia, deltamethrin treated container covers supplemented deltamethrin treated window and door nets after eight months in about half the clusters [42]; and in Mexico, researchers added spinosad treatment of productive water containers to cypermethrin treated door and window screens after 14 months.

The number of clusters randomised to intervention and control status varied widely, from just one very large intervention and one very large control cluster in Brazil [36] to 22 intervention and 66 small control clusters in Thailand [41]. The largest number of households to receive the intervention was also in Thailand, although the researchers only measured entomological indices in half of these [41]. The duration of follow up varied from six weeks to 18 months after the start of an intervention. In the three studies with two-staged interventions, the last measurements of entomological indices were at six weeks [40, 42] to 10 months [43] after the second intervention. For interventions beginning at single time point, the last measurements were at between four months [39] and 18 months [41].

Measured impacts of the interventions varied considerably. The temephos trial found no effect; the BI and CI were slightly lower in control than intervention clusters at most time points [36]. In the trials concerned with insecticide-treated window and door screens or curtains, three found an impact on pupal densities and other indices mainly after addition of the second intervention of treating productive containers [40, 43] or of adding treated container covers [42]. The trial of treated door and window nets alone found that the impact on BI at six months, when 71% of households used the nets, was not maintained at 18 months, when only a third of households used the nets [41]. In the report of the trial of treated window and door nets in Mexico and Venezuela, with added treated container covers in Venezuela only, the authors found a reduction in entomological indices in all clusters, not different between intervention and control clusters, and attributed this to a spill-over into the nearby control clusters. The authors of the Haiti treated bed nets trial also attributed the fall in indices in all clusters, with no difference between intervention and control clusters, to a spill-over effect. The trial of deltamethrin lethal ovitraps and Bti, alone and in combination, with education and household visits as the control condition, found no difference in entomological indices between the intervention clusters and the control cluster. The authors postulated this could be because the initial education and clean-up followed by repeated visits were in themselves an intervention as effective as the interventions being tested [39].

Only two of the CRCTs measured the impact of chemical interventions on dengue infection as well as on entomological indices, with inconclusive findings. In the trial of deltamethrin treated window curtains and container covers in Venezuela, Kroeger et al. reported that positive adult dengue IgM serology at eight months was lower in intervention than control clusters, with borderline statistical significance [37]. In the trial of treated bed nets in Haiti, in all clusters there were fewer individuals positive for dengue IgM at 12 months; the authors considered the lack of difference between intervention and control clusters reflected a spill-over effect.

### Biological control interventions

Only one study of a biological control cluster trial met the inclusion criteria. Kittayapong et al. in Thailand of using either copepods or Bti (the households had a choice) in household water containers to control breeding of the dengue vector [44]. The intervention also included community mobilisation meetings and recruitment of eco-health volunteers (EHVs) from among existing community health volunteers. The EHVs visited households to deliver the biological control materials and educated household members on elimination of vector breeding sites. Public services cleaned up communal spaces in the communities. Although there was also an element of community mobilisation, we categorised this trial as primarily of the biological control methods. The study compared 10 intervention clusters with 10 control clusters, with measurements of vector indices up to six months. The HI, CI and BI were significantly lower at follow up than at baseline in all clusters, but not so in control compared with intervention clusters. The PPI was significantly lower in intervention than control clusters at all time points after the baseline.

### Community mobilisation and participation interventions

We categorised nine CRCTs as primarily trials of community mobilisation and participation, seven from Central and South America [45, 46, 49–53] and two from Asia [47, 48]. One trial from Mexico measured the impact of an educational intervention at household and community level and a chemical intervention (space spraying with malathion and temephos applied to household water containers), alone or in combination, compared with a control cluster with neither intervention [45]. Common features of the complex interventions included: engagement of local stakeholders in discussions of the problems and planning of activities; involvement of community members in prevention and dissemination activities; household visits to support their efforts to reduce dengue breeding sites; educational programmes at household and community levels; partnerships with local services; and efforts to improve local services such as garbage collection. Four trials involved schools and schoolchildren [47, 50, 51, 53] or elders [50]. Two noted the importance of involving women [47, 53]. Specific activities included: distribution of locally made covers for water containers [47], promoting composting of biodegradable waste [48], and collecting small waste items from around houses [52].

In all trials, the routine government dengue control activities continued in the intervention as well as control clusters, so the measured impact was of the community mobilisation in addition to the routine prevention activities. In the trial from Ecuador [51], the analysis was complicated by a change in the government programme midway through the intervention: from a programme based on temephos in water and insecticide space spraying to use of a biolarvicide (Bti) and education for source reduction.

The included CRCTs varied in size, from a very small study in Mexico with three intervention clusters and one control cluster and a total of 187 households [45], and a small study in Sri Lanka with four intervention and four control clusters and 1593 households [48], to a study in Cuba with 16 intervention and 16 control clusters and a total of 19,170 households [46], and a trial in Nicaragua and Mexico with 75 intervention and 75 control clusters and a total of 18,838 households [53]. Length of follow up varied from five months [45, 50, 52] to 24 months [49]. Some trials reported only measurements at baseline and follow up [45, 50–52], while others made one or more measurements in between [46–48, 53]. One trial in Cuba relied on monthly measurements by the government vector control programme [49].

The reported impacts of the CRCTs varied but were broadly positive, with a significant impact on at least one entomological index. Four studies found all the measured indices were significantly lower in the intervention than control clusters at the last follow up [46, 47, 50, 53]. The trial from Sri Lanka with a focus on solid waste management found a significant impact on BI at 15 months and on PPI at all time points [48]. The trial in Cuba that used figures from the routine government surveillance found significantly lower BI in intervention clusters at all time points [49]. The Ecuador trial of the elementary school education programme and the clean patio safe container programme detected a significant impact on PPI only at 12 months, but only when clusters without full implementation were excluded. This trial was complicated by the change (probably improvement) in the government programme in the control sites midway through the intervention [51]. The Uruguay trial reported a non-significant difference between intervention and control cluster in favour of the intervention; low vector densities in the sites reduced the power of the study to detect significant differences [52]. The small complicated trial from Mexico compared an educational intervention, with or without malathion spraying, with a control cluster. It found a significant impact of the education programme only on a specific index (positive containers per household); this impact was less marked when the education intervention was combined with malathion space spraying [45].

Only one CRCT of community mobilisation measured the impact on dengue infection. The trial in Nicaragua and Mexico found a significant impact on childhood dengue infection (assessed by dengue antibodies in paired saliva samples) and on self-reported dengue cases in households [53].

### Meta-analysis

We assessed six studies in the meta-analysis as having a “low risk of bias” and four as having an “unclear risk of bias”, because they did not report some of the information needed to assess elements of the risk of bias (Table 3).

Eight of ten articles in the meta-analysis provided the necessary data to calculate the combined effectiveness for all three entomological indices (HI, CI and BI). One study provided information for only two indices (HI and BI) and one provided information only for the HI. Table 4 summarizes the data for the *Aedes aegypti* indices in the last measurement for each study’s intervention and control groups, with calculated intervention effectiveness estimates (RD and 95% CI). In every trial of community participation, the estimated intervention effectiveness was positive, showing a decrease in the HI, CI and BI; the higher 95% CI limit for these estimations is 0.03 (for the HI and BI), from the study by Basso et al. in Uruguay [52].

**Table 4:** Intervention effectiveness on dengue vector control of studies in meta-analysis

First author & year	Time to impact measurement (months)	Intervention type	Parameters	Intervention clusters	Control clusters	Intervention effectiveness (RD and 95%CI)
Kroeger (2006)	Mexico 9 Venezuela 12	Chemical control	HI CI BI	0.09 0.01 0.11	0.12 0.02 0.14	-0.03 (−0.06; 0.00) −0.01 (−0.02; 0.00) −0.03 (−0.06; 0.00)
Lenhart (2008)	5	Chemical control	HI CI BI	0.05 0.02 0.06	0.03 0.01 0.03	0.02 (−0.01; 0.05) 0.01 (0.00; 0.19) 0.03 (0.00; 0.06)
Ocampo (2009)	15	Chemical control	HI	0.00	0.05	−0.05 (−0.10; 0.00)
Vanlerberghe (2013)	12	Chemical control	HI CI BI	0.14 0.66 0.22	0.19 0.55 0.24	−0.05 (−0.09; −0.01) 0.11 (−0.04; 0.19) −0.02 (−0.06; 0.02)
Quintero (2015)	8	Chemical control	HI CI BI	0.07 0.02 0.07	0.03 0.01 0.03	0.04 (0.02; 0.07) 0.01 (0.00; 0.02) 0.04 (0.02; 0.07)
Kittayapong (2012)	8	Biological control	HI CI BI	0.12 0.03 0.25	0.14 0.05 0.33	−0.02 (−0.07; 0.03) −0.02 (−0.04; −0.01) −0.08 (−0.15; −0.01)
Vanlerberghe (2009)	12	Community participation	HI BI	0.26 0.28	0.48 0.52	−0.22 (−0.23; −0.21) −0.24 (−0.25; −0.23)
Arunachalam (2012)	18	Community participation	HI CI BI	0.04 0.01 0.04	0.16 0.06 0.21	−0.12 (−0.15; −0.09) −0.05 (−0.06; −0.04) −0.17 (−0.20; −0.14)
Basso (2015)	6	Community participation	HI CI BI	0.07 0.07 0.12	0.07 0.08 0.14	0.00 (−0.03; 0.03) 0.00 (−0.03; 0.02) −0.01 (−0.06; 0.03)
Andersson (2015)	Nicaragua 32 Mexico 32	Community participation	HI CI BI	0.14 0.05 0.20	0.20 0.08 0.30	−0.06 (−0.07; −0.05) −0.03 (−0.03; −0.02) −0.10 (−0.12; −0.09)

*HI* = household index; *CI* = container index; *BI* = Breteau index

**Fig. 2:**
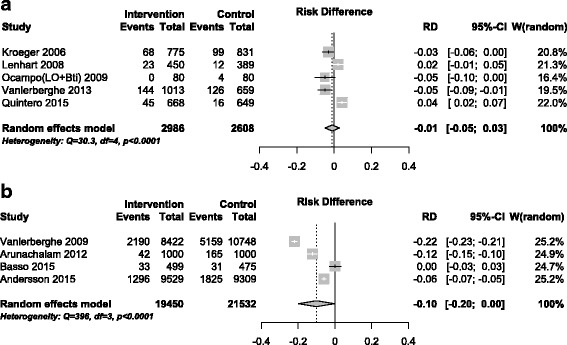
Intervention effect: Household Index; **a** Chemical control studies; **b** Community participation studies

The overall intervention impact assessments for the *Household Index* were −0.01 (95% CI -0.05 to 0.03) for chemical control, and −0.10 (95% CI -0.20 to 0.00) for community participation (Fig. 2). None of the confidence intervals for impact on HI from the studies of community participation interventions included unity, reflecting a consistently significant impact on this index. The single CRCT of biological control reported an impact of −0.02 (95% CI -0.07 to 0.03) on the HI.

**Fig. 3:**
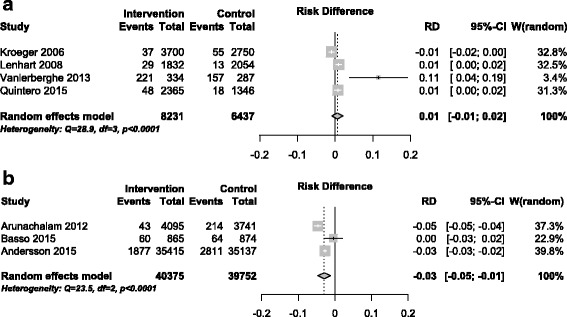
Intervention effect: Container Index. **a** Chemical control studies; **b** Community participation studies

For the *Container Index*, community participation interventions again showed the most consistent impact. The overall intervention impact assessments for CI were 0.01 (95%CI -0.01 to 0.02) for chemical control interventions, and −0.03 (95%CI -0.05 to −0.01) for community participation interventions (Fig. 3). The single CRCT of biological intervention reported an impact of −0.02 (95%CI -0.04 to −0.01) on the CI.

**Fig. 4:**
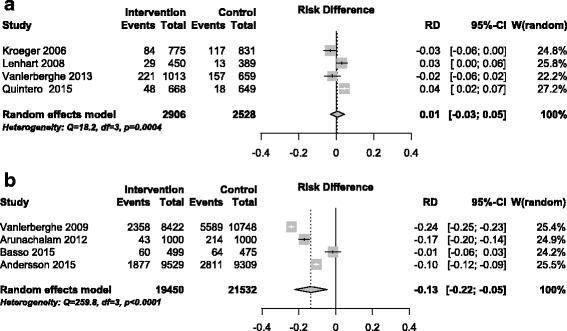
Intervention effect: Breteau Index. **a** Chemical control studies; **b** Community participation studies

The estimated combined intervention impact of chemical control on the *Breteau Index* was 0.01 (95% CI -0.03 to 0.05), while that of community participation was −0.13 (95% CI -0.22 to −0.05) (Fig. 4). The impact on BI of the single biological control trial was −0.08 (95% CI -0.15 to −0.01).

We found significant heterogeneity (p -value <0.001) between the included studies for each of the entomological indices (HI, CI, BI) for both chemical control CRCTs and community mobilisation CRCTs. For both chemical control studies and community mobilisation studies, sensitivity analysis showed that no study, when it was excluded from the meta- analysis, substantially changed the overall outcome for any of the indices. We found no statistical evidence of publication bias; all the *p* values obtained from the Begg and Egger test were 0.10 or greater, and nearly all were greater than 0.18.

## Discussion

This systematic review and meta-analysis of 18 CRCTs published between 2002 and 2015 suggests that community mobilisation programmes are an effective intervention to reduce *Aedes aegypti* entomological indices.

An earlier systematic review by Erlanger and colleagues included multiple types interventions (chemical, biological, and community-based), concluding that integrated interventions including community involvement were the most effective [15]. Ballenger-Browning and Elder concluded that the evidence base was not good enough to draw conclusions [17]. Our findings are similar to those reported by Bowman in 2016, where community based interventions for dengue vector control showed higher impact than those using insecticide-treated curtains [25]. The four CRCTs of community participation in our review reported continuation of government vector control (usually temephos application and area fumigation) in both intervention and control sites [46, 47, 52, 53]. The observed decreases in the HI, CI and BI represent added effectiveness from community mobilisation.

Only one CRCT of a biological control intervention met our inclusion criteria. The Thailand trial of copepods or *Bti*, together with some community activation, reported no significant difference in entomological indices between intervention and control communities at six months [44]. Biological control is attractive as it avoids chemical contamination to the environment, but it may have operational limitations for large scale application. Erlanger noted that biological control has only been tested on a small scale [15] and Bowman noted the clear need for adequately sized CRCTs of biological control interventions for dengue prevention [25].

### Strengths and weaknesses

Unlike earlier systematic reviews [14–25], our review only included CRCTs. Our meta-analysis required data for calculating classic *Aedes aegypti* entomological indices. This limited the number of studies eligible to be included but it meant that the quality of the included studies was relatively good. None of the 18 studies included in our systematic review was considered to have a high risk of bias, although 10 had an “unclear” risk of bias, mostly due to lack of information in the reports. Other limitations of our meta-analysis are the heterogeneity of intervention duration, the small number of clusters in some of the studies, and the variable cluster size, all of which could affect the intervention effectiveness estimates. The sensitivity analysis, however, showed stability of the global effectiveness estimates.

The grouping of different kinds of interventions together into the broad categories of chemical interventions, biological interventions, and community mobilisation interventions in the meta-analysis could lead to the effectiveness of a particular intervention being under-estimated because it is over-shadowed by poor performance of other interventions in the same broad group. We do not believe this is likely in our study. The four community mobilisation studies all showed positive impacts, albeit of varying magnitude. And among the five chemical intervention trials, three were of treated window and door curtains or nets, and one was of treated bednets, with only one being a different type of intervention (lethal ovitraps).

We were not able to include the main chemical control methods used in government *Aedes aegypti* control programmes – temephos in domestic water containers and peri-domestic insecticide spraying – in our meta-analysis because we did not identify any CRCTs with details of impact on entomological indicators. In the descriptive review, we included one CRCT of temephos use, with no significant impact on entomological indices [36], and a CRCT that studied both and education programme and ultra-low volume malathion spraying and temephos application, and found that the ULV spraying *reduced* the effectiveness of the educational intervention [45].

Table 3 and Figs. 2- 4 are based on the last measurement point comparing intervention and control sites in each trial. It is possible that this missed some useful impact for some of the interventions. In the trials reported by Lenhart et al. [38] and Vanlerberghe et al. [41] the difference between intervention and control clusters was greater in earlier measurements than later measurements; the authors attributed this to spill-over effects or reduced coverage of the treated materials over time.

### Public health implications

Cluster trials, assessing community effectiveness, unlike household or container based trials, take account of community level dynamics. In this real life setting, our review shows chemical control was less effective than community mobilisation, for all three entomological indices.

Depositing temephos in water storage containers is the mainstay of most centrally managed *Aedes aegypti* control programmes in Latin America and elsewhere [6]. A recent systematic review of the effectiveness of temephos for dengue vector control concluded there was evidence of impact on entomological indices of *Aedes aegypti* when temephos use was evaluated as a single intervention; effectiveness varied considerably depending on factors such as frequency and method of application and usually did not persist for more than three months. The effect of temephos was less in studies where temephos was part of a combined intervention, as it is almost everywhere in *Aedes aegypti* control programmes [21]. Most of the studies in the temephos review by George et al. were not CRCTs [21]. The single CRCT of the use of temephos alone included in our systematic review reported no impact of temephos on entomological indices [36]. In Guatemala, the use of temephos together with deltamethrin treated window and door nets had an impact on some, but not all, entomological indices [40].

Outside the research context, *Aedes aegypti* control almost everywhere implies complex interventions and cluster dynamics. Community mobilisation implies changes in human attitudes and behaviour, which in turn has multiple effects: people might be motivated to control breeding sites and to cover water containers, to work together on communal vector breeding sites like cemeteries, and they might also be motivated to remove pesticide from water containers. From a centrally managed programme, it would be difficult to foresee the exact mix of interventions to suit every community. Centrally managed awareness and education programmes are thus a weak basis to achieve community commitment to and ownership of interventions. Sustainable community engagement includes local evaluation of evidence and co-designing interventions that best suit their local conditions and culture [54]. This community authorship, rather than interventions being imposed or advised from outside, seems to underwrite the success of the Camino Verde intervention in Mexico and Nicaragua [55].

The cost implications of multi-faceted programmes for vector control need further study. Countries using temephos and insecticide spraying as key elements of national vector control programmes already carry the expense of centralised programming and logistical structures, and the vertical management and huge numbers of local personnel required to achieve monthly or bimonthly household visits. These countries are paying for vector control that, judging by the relentless increase in dengue risk and recent explosive zika and chikungunya epidemics, does not work very well. A central concern in adding community engagement efforts is how much this would add to effectiveness and acceptability, in relation to the added cost. The cost of adding community engagement might also be offset if it helped to support uptake of a dengue vaccine as that becomes a real public health option.

## Conclusion

The implications of our review for dengue vector control are clear. The most consistently effective intervention was community mobilization. Governments that rely on chemical control of *Aedes aegypti* should consider adding community mobilization to their prevention efforts.

More well-conducted CRCTs of complex interventions, including those with biological control, are needed to provide evidence of real life impact. Future trials of interventions of all kinds should include measurement of impact on dengue infection as well as on entomological indices.

## Abbreviations

95%CI: 95% confidence intervals
BI: Breteau index
CI: Container index
HI: Household index
PHI: Pupae per household index
PPI: Pupae per person index
RD: Risk difference
SEPA: Socialisation of evidence for participatory action
